# Maternal Asian ethnicity and obstetric intrapartum intervention: a retrospective cohort study

**DOI:** 10.1186/s12884-016-1187-2

**Published:** 2017-01-05

**Authors:** Maya Reddy, Euan M. Wallace, Joanne C. Mockler, Lynne Stewart, Michelle Knight, Ryan Hodges, Sasha Skinner, Miranda Davies-Tuck

**Affiliations:** 1 Monash Health, Monash Medical Centre, Clayton, Australia; 2 The Ritchie Centre, Hudson Institute of Medical Research, Clayton, Australia; 3 Department of Obstetrics and Gynecology, Monash University, Level 5, Monash Medical Centre, Clayton, VIC 3168 Australia

**Keywords:** Asian ethnicity, Cesarean section, Intrapartum outcomes, Operative vaginal birth

## Abstract

**Background:**

Maternal ethnicity is a recognized risk factor for stillbirth, such that South Asian women have higher rates than their Caucasian counterparts. However, whether maternal ethnicity is a risk factor for intrapartum outcomes is less clear. The aim of this study is to explore associations between maternal country of birth, operative vaginal delivery and emergency cesarean section, and to identify possible mechanisms underlying any such associations.

**Methods:**

We performed a retrospective cohort study of singleton term births among South Asian, South East/East Asian and Australian/New Zealand born women at an Australian tertiary hospital in 2009–2013. The association between maternal country of birth, operative vaginal birth and emergency cesarean was assessed using multivariate logistic regression.

**Results:**

Of the 31,932 births, 54% (17,149) were to Australian/New Zealand-born women, 25% (7874) to South Asian, and 22% (6879) to South East/East Asian born women. Compared to Australian/New Zealand women, South Asian and South East/East Asian women had an increased rate of both operative vaginal birth (OR 1.43 [1.30–1.57] and 1.22 [1.11–1.35] respectively, *p* < 0.001 for both) and emergency cesarean section (OR 1.67 [1.53–1.82] and 1.16 [1.04–1.26] respectively, *p* < 0.001 and *p* = 0.007 respectively). While prolonged labor was the predominant reason for cesarean section among Australian/New Zealand and South East/East Asian women, fetal compromise accounted for the majority of operative births in South Asian women.

**Conclusion:**

South Asian and South East/East Asian women experience higher rates of both operative vaginal birth and cesarean section in comparison to Australian/New Zealand women, independent of other risk factors for intrapartum interventions.

## Background

Maternal ethnicity is known to be an independent risk factor for a variety of adverse pregnancy and perinatal outcomes. In particular, women of Asian ethnicity giving birth in high-income countries exhibit higher rates of adverse pregnancy outcomes than other women accessing the same health services [1–7]. For example, Asian women have three times the rate of gestational diabetes than Caucasian women [1, 2]. Migrant Asian women are also more likely to have a small for gestational age (SGA) infant, preeclampsia, and spontaneous and iatrogenic preterm birth [1, 3–5]. Furthermore, South Asian (SA) women have been consistently shown to have a higher rate of stillbirth than Caucasian women, particularly at or post-term [4], independent of socioeconomic status, maternal support, and pre-existing medical or pregnancy conditions [1, 3, 4, 6, 7].

While the increased rates of adverse antenatal outcomes for SA women compared to other women are consistent observations, studies exploring the relative rates of various intrapartum interventions, such as cesarean section and operative vaginal birth rates among women of different ethnicities are less so [8–11]. Some authors have reported that rates of intrapartum cesarean section do not differ between women of varying ethnicities [8, 9, 12] while others have observed higher rates of cesarean section and operative vaginal delivery amongst some Asian groups [11]. Furthermore, few studies have examined the indication for operative delivery between different ethnic groups [13]. Accordingly, we undertook this study to clarify whether maternal Asian ethnicity is associated with intrapartum interventions and if so, to explore the indications and likely mechanisms underlying any such differences.

## Methods

We studied all singleton births ≥37 weeks gestation and free from congenital anomalies at Monash Women’s Services, Monash Health, a metropolitan university teaching hospital in Melbourne, Australia, from 2009 to 2013 inclusive. Data were extracted from the Birthing Outcomes System (BOS), an electronic database recording all births ≥20 weeks’ gestation. For each birth the attending midwife, supported by routine data validation, enters 46 data items into BOS [14]. For this study, we extracted data from the following fields: maternal age, body mass index (BMI), country of birth, parity, maternal and obstetric medical conditions, smoking, whether women had a first trimester ultrasound, gestation at birth, birthweight, identified need for an interpreter, onset of labor (spontaneous or induced), augmentation of labor, epidural use, operative vaginal birth, length of first and second stage of labor, private or public care, baby gender, mode of delivery and indication for surgical delivery. Fields were largely complete. Data was missing for the following fields only; maternal BMI (0.8%), birth weight (0.03%), length of first stage (0.003%), length of second stage (0.003%), parity (0.003%), first trimester ultrasound (0.009%) and onset of labour (0.003%), all other data were complete. Missing data were case-wise excluded. This study was granted an exemption from ethics review by the Monash University Human Research and Ethics Committee as per section 5.1.22 of the National statement on ethical conduct in Human Research 2007 (ID 14143B 15/5/2014).

Women were classified into regional groups, as a surrogate measure of ethnicity, according to their self-reported country of birth as defined by the United Nations [15]. Briefly, women were defined as being from Australia or New Zealand, South Asia (e.g. Afghanistan, Bangladesh, Bhutan, India, Iran, Maldives, Nepal, Pakistan and Sri Lanka) and South East and East Asia (e.g. Brunei Darussalam, Cambodia, Indonesia, Laos, Malaysia, Myanmar, Philippines, Singapore, Thailand, Timor-Leste and Vietnam, China, Hong Kong, Macao, North Korea, South Korea, Japan, and Mongolia). Women of other nationalities were excluded because the aim of this study was to examine comparative outcomes in Asian women.

### Statistical analyses

Maternal demographics, antenatal and intrapartum factors were tabulated by maternal country of birth. Differences across groups were determined by a chi^2^ or Kruskal-Wallis test. The rates of spontaneous vaginal birth, operative vaginal birth, planned cesarean and emergency cesarean were also determined for each risk factor. Logistic regression was used to examine the association between maternal country of birth and spontaneous vaginal birth, operative vaginal birth and planned and intrapartum cesarean delivery. Known and potential risk factors that were assessed for their inclusion in the final model were maternal age, BMI, parity, need for interpreter, labor type (spontaneous, induced or augmented), epidural, birth weight over 4000 g, private patient, length of first and second stage of labor, past cesarean, first trimester ultrasound, pre-existing hypertension, pre-existing diabetes, gestational hypertension/pre-eclampsia/HELLP, gestational age, baby gender, and birthweight <10^th^ centile (birthweight centiles were not customized for ethnicity). The association between maternal country of birth, spontaneous vaginal birth, operative vaginal birth, and planned and emergency cesarean, adjusted for confounders, was then assessed using stepwise multivariable logistic regression, including only confounders significant at the univariate level. The likelihood ratio was used to determine the final model. Not unexpectedly, the rates of operative vaginal birth and cesarean delivery differed by parity. Therefore, we also performed the regression stratified by parity. Doing so did not change the associations between maternal country of birth and the outcomes and so we have presented data for all women regardless of parity. The indications for intrapartum cesarean section and operative vaginal birth by maternal country of birth group were also tabulated. A *p*-value <0.05 (two-tailed) was regarded as significant. All analyses were performed using the SPSS statistical package (SPSS 20, IBM Corp, Armonk, New York, USA).

## Results

Between 2009 and 2013 there were 41,040 singleton births at the maternity service. Of these, 31,932 (78%) births were at term to women from one of the three regions of origin. Of these, 17,149 (54%) births were to AUS/NZ born women, 7874 (25%) to SA born women, and 6879 (22%) to SE/EA born women. The characteristics of the women are presented in Table 1. Compared to AUS/NZ women, women from SA and SE/EA were older, had a lower BMI, were less likely to smoke, were less likely to access private care, were more likely to be nulliparous and had lower rates of pre-existing hypertension. Women from SA and SE/EA were also more likely to develop gestational diabetes compared to AUS/NZ women. Women from SE/EA were less likely to have pre-existing diabetes or past history of cesarean section. Hypertensive disorders of pregnancy were more common in the AUS/NZ born women. About 10% of babies born to AUS/NZ mothers were considered small-for-gestational age (SGA <10^th^ centile) in comparison to 16.6% of those born to SA and 13% of those born to SE/EA.

**Table 1:** Characteristics by maternal country of birth (*n* = 34,721)

	Australia/NZ	South Asia	South East Asia	*P* value
	(*n* = 17149)	(*n* = 7874)	(*n* = 6879)	
Maternal age groups				
< 20 years	687(4.0%)	37(0.5%)	43(0.6%)	<0.001
20–30years	7476(43.5%)	4181(53.1%)	2787(40.5%)	
> 30 years	9016(52.5%)	3656(46.4%)	4049(58.9%)	
Maternal BMI (kg/m^2)^				
< 19	561(3.3%)	461(5.9%)	798(11.7%)	<0.001
19–24.9	7121(41.9%)	4161(53%)	4716(69.1%)	
25–29.9	4667(27.4%)	2404(30.6%)	1073(15.7%)	
≥ 30	4662(27.4%)	820(10.5%)	240(3.5%)	
Nulliparous	7300(42.5%)	3914(49.7%)	3247(47.2%)	<0.001
Interpreter required	88(0.5%)^a^	1479(18.8%)	2441(35.5%)	<0.001
Private patient	2946(17.1%)	602(7.6%)	653(90.5%)	<0.001
Smoker	2797(16.3%)	24(0.3%)	75(1.1%)	<0.001
Previous cesarean	2341(13.6%)	1073(13.6%)	711(10.3%)	<0.001
First trimester ultrasound	14,926(86.9%)	6549(83.2%)	5481(79.7%)	<0.001
Pre-existing hypertension	230(1.3%)	36(0.5%)	29(0.4%)	<0.001
Pre-existing diabetes	181(1.1%)	81(1.0%)	39(0.6%)	0.001
Pre-existing thyroid disease	384(2.2%)	435(5.5%)	141(2.0%)	<0.001
Gestational diabetes	826(4.8%)	892(11.3%)	778(11.3%)	<0.001
Gestational Hypertension/Pre-eclampsia/HELLP	918(5.3%)	242(3.1%)	147(2.1%)	<0.001
Labor induced	4100(23.9%)	1841(23.3%)	1067(15.5%)	<0.001
Labor augmented	3286(19.1%)	1810(23%)	1714 (24.9%)	<0.001
Epidural	3626(21.1%)	1396(17.7%)	1015(14.8%)	<0.001
Length of 1^st^ stage of Labor (hours)^b^	4.3(1.8–8.0)	5.3(2.3–9.2)	5.1(2.4–9.2)	<0.001
Length of 2^nd^ stage of Labor (mins)^b^	15(0–48)	19(0–59)	20(4–58)	<0.001
Gestational age				
37–41 + 6 weeks	16,851(98.1%)	7760(98.6%)	6794(98.8%)	<0.001
≥ 42 weeks	328(1.9%)	114(1.4%)	85(1.2%)	
Small for gestational age <10^th^ centile	1592(9.3%)	1266(16.1%)	896(13%)	<0.001
Birthweight of baby (g)	3491(498)	3309(460)	3312(426)	<0.001
Baby admission to NICU	146(0.8%)	62(0.8%)	44(0.6%)	<0.001
Apgar < 7 at 5 min	292(1.7%)	125(1.6%)	85(1.2%)	0.03
Fetal Compromise in Labour	4231(24.6%)	2369(30.1%)	1588(23.1%)	<0.001

^a^10 Australian women were deaf and required Auslan interpreters

^b^Length of first and second stage – median and interquartile range (25^th^-75^th^ centile)

Regarding labor, women born in SE/EA were less likely to be induced (15.5%) than either AUS/NZ (23.9%) or SA (24.9%) women (*p* < 0.001). However, labor augmentation was more common in SE/EA (24.9%) and SA (23%) women than in AUS/NZ born mothers (19.1%) (*p* < 0.001). Use of epidural anesthesia was greatest in the AUS/NZ born population (21.1%). Overall, Asian women experienced a longer 1^st^ and 2^nd^ stage of labor compared to AUS/NZ women (*p* < 0.001 for both). APGAR scores at delivery were similar across ethnic groups. However admission rates to NICU were similar between the SA and AUS/NZ populations and lower in the SE/EA born women (*p* < 0.001).

The rate of spontaneous vaginal delivery differed amongst ethnic groups (Table 2) and was highest amongst SE/EA (61.8%) and AUS/NZ (59.4%) born women and lowest in the SA population (51.8%). After adjusting for confounding variables this conferred a 50% (95% CI; 0.46–0.54) decreased odds of spontaneous vaginal delivery for SA women compared to AUS/NZ women (*p* < 0.001) and a 24% (95% CI 0.69–0.84) decreased odds for SE/EA women (*p <* 0.001). The rate of planned cesarean section was comparable between SA and AUS/NZ women (OR1.02, 95% CI (0.91–1.14). However SE/EA born women had a significantly lower rate of planned cesarean section (OR 0.87, 95% CI (0.76–0.98) (*p* = 0.02).

**Table 2:** Maternal country of birth and intrapartum obstetric intervention

	Prevalence of intervention	Crude odds ratio (95% CI)	*P* value	Adjusted odds ratio (95% CI)	*P* value
Spontaneous vaginal birth					
Australia/NZ	10,212(59.4%)	1	-	1	-
South Asia	4075(51.8%)	0.73(0.69, 0.77)^a^	<0.001	0.50(0.46,0.54)^b^	<0.001
South East-East Asia	4249(61.8%)	1.10(1.04,1.17)^a^	0.001	0.76(0.69,0.84)^b^	<0.001
Operative vaginal birth					
Australia/NZ	2235(13%)	1	-	1	-
South Asia	1327(16.9%)	1.36(1.26,1.50)^a^	<0.001	1.43(1.3,1.57)^b^	<0.001
South East-East Asia	1004(14.6%)	1.14(1.05,1.24)^a^	<0.001	1.22(1.11,1.35)^b^	<0.001
Intrapartum cesarean					
Australia/NZ	2325(13.5%)	1	-	1	-
South Asia	1460 (18.5%)	1.45(1.31,1.51)^c^	<0.001	1.67(1.53,1.82)^d^	<0.001
South East-East Asia	913(13.3%)	0.95(0.87,1.03)^c^	0.22	1.16(1.04,1.26)^d^	0.007
Planned cesarean					
Australia/NZ	2383(13.9%)	1	-	1	-
South Asia	1000(12.7%)	0.90(0.83,0.98)^e^	0.01	1.02(0.91,1.14)^f^	0.73
South East-East Asia	703(10.2%)	0.71(0.65,0.77)^e^	<0.001	0.87(0.76,0.98)^f^	0.02

^a^Odds ratio of having a spontaneous vaginal delivery or operative vaginal birth with respect to maternal country of birth

^b^Odds ratio of having an spontaneous vaginal delivery or operative vaginal birth after adjustment for all variables significant at the univariate level. These variables included maternal age group, BMI, parity, baby birthweight, private care, length of first stage, length of second stage, induction of labor, augmentation of labor and use of epidural

^c^Odds ratio of having an emergency cesarean birth with respect to maternal country of birth

^d^Odds ratio of having an emergency cesarean birth after adjustment for all variables significant at univariate level. These variables included maternal age group, maternal BMI, parity, past cesarean, pre-existing diabetes, gestational hypertension/PE/HELLP, gestational age, baby gender, baby birthweight, private patient, prolonged first stage, labor induced, labor augmented and use of epidural

^e^Odds ratio of having a planned cesarean section with respect to maternal country of birth

^f^Odds ratio of having an planned/elective cesarean birth after adjustment for all variables significant at univariate level. These variables included maternal age group, maternal BMI, parity, past cesarean, baby gender and private patient

The rate of operative vaginal birth differed significantly by maternal country of birth. The rate of operative vaginal birth was highest in SA women (16.9%), followed by SE/EA women (14.6%) and then AUS/NZ women (13%), representing a 36% (95% CI; 1.26–1.50) increased odds of operative vaginal birth for SA women compared to AUS/NZ women (*p* < 0.001) and an 14% (95% CI; 1.05–1.24) increased odds for SE/EA women (*p* < 0.001). After adjustment for confounders, compared to AUS/NZ born women, SA women had a 43% (95% CI; 1.3–1.57, *p* < 0.001) and SE/EA women a 22% (95% CI; 1.11–1.35, *p* < 0.001) increased odds of operative vaginal birth (Table 2). For all groups, fetal compromise was identified as the principal indication for operative vaginal birth and was highest among AUS/NZ (54%), followed by SA (49.6%) and then SE/EA (47.6%) women (*p* = 0.003). A prolonged labor was the most frequent indication for an operative vaginal birth among SE/EA women (42.4%) followed by SA (38%) and AUS/NZ (36.1%) women (*p* = 0.006).

The rate of intrapartum cesarean also differed significantly by maternal country of birth. The rate of emergency cesarean was significantly higher in SA women (18.5%) than in either AUS/NZ(13.5) or SE/EA(13.3) women (*p* < 0.001). After adjusting for confounders, SA women had a 67% increased odds (95% CI; 1.53–1.82, *p* < 0.001) and SE-EA women a 16% increased odds (95% CI; 1.04–1.26) of intrapartum cesarean section, compared to AUS/NZ women (*p* = 0.007). The primary indication for intrapartum cesarean section also varied significantly between maternal country of birth. Fetal compromise was most common in SA women (42%) followed by AUS/NZ (36%) and then SE/EA (26.3%) women (*p* < 0.001). In contrast, prolonged labor was the most common indication in SE/EA (65%), followed by AUS/NZ (57%) and then SA (55%) women (*p* < 0.001).

## Discussion

Among women having a singleton birth in our health service, maternal country of birth was an independent risk factor for both operative vaginal birth and intrapartum cesarean section. Furthermore, the principal indications for both interventions differed by maternal country of birth. In particular, fetal compromise was a more common indication for intrapartum cesarean section in South Asian women than other women. These observations expand on the growing body of evidence that maternal country of birth, or maternal ethnicity, should be recognized as a risk factor for fetal compromise and intrapartum obstetric interventions.

Previous studies that failed to observe a relationship between ethnicity and intrapartum intervention have commonly defined the diverse Asian population as a single entity [8, 9]. However, when Asian women are defined by their region or country of birth, differences in intrapartum outcomes are apparent [11, 16–18]. Consistent with our findings, this point was also illustrated in a meta-analysis of migration and cesarean birth, which showed that South Asian women have an increased risk of cesarean section in comparison to the non-migrant population, while South East/East Asians have a more modest difference in intrapartum outcomes [13]. Furthermore, other studies examining the South Asian population in England and New Zealand have shown that these women have higher rates of operative delivery compared to their Caucasian counterparts. We, and others [19, 20], have also found that both SA and SE/EA women have an increased rate of operative vaginal birth.

We were interested to explore not just differences in rates of intervention between ethnic groups, but also possible mechanisms underlying any observed differences. It is possible that differing prevalence of known risk factors for obstetric intervention such as social deprivation, poor access to healthcare, communication difficulties and pre-existing medical conditions, such as gestational diabetes mellitus (GDM) may explain ethnic differences in Asian women. We were able to adjust for these and, consistent with other populations [5, 16–18, 21–23], showed that such possible confounders could not account for differences in outcomes.

We were also able to show that the indications for operative births varied between ethnic groups. Specifically, the increased rate of cesarean section among SA women was largely due to an increased rate of intrapartum fetal compromise. This confirms previous studies [20, 23] and is in accord with the increased rate of both meconium stained liquor and late pregnancy stillbirth in SA women compared to other women [1, 4]. Taken together, these studies suggest that, on average, the term fetus of a SA woman is relatively “high risk” and that interventions to reduce perinatal morbidity and mortality in this group may need to be considered at earlier term gestations than in other women. In that regard, guidelines for post-term and intrapartum fetal surveillance do not identify maternal ethnicity as a risk factor or indication for earlier induction of labor or for continuous intrapartum electronic fetal monitoring [24–27]. Perhaps they should. The mechanisms underlying the increased rates of fetal compromise in SA women are also unclear. It is possible that there are biological differences in placental function and maturation. Supporting this is the observation that, on average, the pregnancies of SA women are of shorter length than those of other women, consistent with earlier maturation [4, 28, 29]. Further research into what underlies these ethnicity-related differences in pregnancy outcomes is certainly warranted. Such insights may offer opportunities for targeted reductions in perinatal mortality and morbidity in all women. Interestingly, the primary indication for operative vaginal delivery in SA women was not fetal compromise. It is likely that this reflects earlier intervention resulting in intrapartum cesarean for fetal compromise in SA women, thus resulting in fewer women requiring second stage intervention. In contrast, the increased rate of cesarean section among SE/EA women was largely secondary to prolonged or obstructed labor. The reasons for that, and the increased need for labor augmentation in SE/EA women, remain obscure. A more detailed analysis of labor progress and management in SE/EA women compared to Caucasian women would be of interest.

Whatever the underlying reasons for increased intervention rates, both operative vaginal birth and cesarean section are associated with significant maternal and perinatal morbidity. Operative vaginal births are associated with higher rates of both maternal and fetal trauma as well as postpartum hemorrhage [30–34]. Cesarean deliveries are associated with a higher rate of hemorrhage, endometritis, venous thromboembolism and longer recovery [35]. These complications happen with greater frequency during emergency cesarean sections [35]. In future pregnancies, a primary cesarean section is also associated with higher rates of abnormal placentation, and uterine rupture [36] and is the major indication for a further cesarean section [37–40]. A better understanding of the mechanisms by which maternal ethnicity increases the need for obstetric intervention may inform better care and, in turn, increase normal vaginal birth rates.

Our study has a number of limitations. As a retrospective analysis drawn from a single center, the demographic of the population and the predispositions of physician practice may all have influenced mode of delivery and so limit generalizability. We don’t believe this to be likely because our broad findings are in accord with those of others. However, it would be useful for other centers to explore whether indications for cesarean section differed between maternal ethnic groups. Of course, the indication recorded for any operative delivery is dependent on the attending accoucher and therefore inter-practitioner variability is possible. Nonetheless, we believe that the large number of practitioners that would be represented in our study makes our findings more, and not, less generalizable. In this study, we were only able to define ethnicity by maternal country of birth and as such it is possible that some women within the AUS/NZ born group are of Asian ethnicity. However, we believe that this is more likely to have underestimated rather than overestimated the associations. Nonetheless, we would call for all jurisdictions to record both maternal ethnicity and maternal country of birth rather than one or the other. Lastly, non-independence is a recognized issue when using population based perinatal datasets. We assessed our associations in both nulliparous and parous women. Doing so did not substantially change our findings.

## Conclusion

Maternal country of birth is an independent risk factor for operative vaginal birth and emergency cesarean delivery. In regards to emergency cesarean section, the principal indications for surgery also differ by maternal country of birth with fetal compromise being more common in SA women and lack of progress of labor being more common in SE/EA women. Understanding these differences further is likely to improve maternity care for all women.

## Abbreviations

AUS/NZ: Australian/New Zealand
SA: South Asian
SE/EA: South East/East Asian
